# Astrocyte activation in hindlimb somatosensory cortex contributes to electroacupuncture analgesia in acid-induced pain

**DOI:** 10.3389/fneur.2024.1348038

**Published:** 2024-04-03

**Authors:** Qing Ye, Jie Li, Wen-Jing Ren, Ying Zhang, Tao Wang, Patrizia Rubini, Hai-Yan Yin, Peter Illes, Yong Tang

**Affiliations:** ^1^International Joint Research Centre on Purinergic Signalling, School of Acupuncture and Tuina, Chengdu University of Traditional Chinese Medicine, Chengdu, China; ^2^Hospital of Chengdu University of Traditional Chinese Medicine, Chengdu, China; ^3^Chongqing Traditional Chinese Medicine Hospital, Chongqing, China; ^4^Rudolf Boehm Institute of Pharmacology and Toxicology, University of Leipzig, Leipzig, Germany; ^5^Acupuncture and Chronobiology Key Laboratory of Sichuan Province, School of Health and Rehabilitation, Chengdu University of Traditional Chinese Medicine, Chengdu, China

**Keywords:** electroacupuncture analgesia, astrocytes, acid-induced pain, hindlimb somatosensory cortex, extracellular acidification

## Abstract

**Background:**

Several studies have confirmed the direct relationship between extracellular acidification and the occurrence of pain. As an effective pain management approach, the mechanism of electroacupuncture (EA) treatment of acidification-induced pain is not fully understood. The purpose of this study was to assess the analgesic effect of EA in this type of pain and to explore the underlying mechanism(s).

**Methods:**

We used plantar injection of the acidified phosphate-buffered saline (PBS; pH 6.0) to trigger thermal hyperalgesia in male Sprague–Dawley (SD) rats aged 6–8 weeks. The value of thermal withdrawal latency (TWL) was quantified after applying EA stimulation to the ST36 acupoint and/or chemogenetic control of astrocytes in the hindlimb somatosensory cortex.

**Results:**

Both EA and chemogenetic astrocyte activation suppressed the acid-induced thermal hyperalgesia in the rat paw, whereas inhibition of astrocyte activation did not influence the hyperalgesia. At the same time, EA-induced analgesia was blocked by chemogenetic inhibition of astrocytes.

**Conclusion:**

The present results suggest that EA-activated astrocytes in the hindlimb somatosensory cortex exert an analgesic effect on acid-induced pain, although these astrocytes might only moderately regulate acid-induced pain in the absence of EA. Our results imply a novel mode of action of astrocytes involved in EA analgesia.

## 1 Introduction

Pain is an unpleasant signal that is associated with tissue damage and involvement of different brain structures, some of which are part of the pain matrix, including the primary somatosensory cortex (S1), primary motor and supplementary motor cortices, secondary somatosensory cortex, anterior cingulate cortex, insular cortex, prefrontal cortex, thalamus, amygdala, and hippocampus (1–9). Each of these regions plays a distinct role in different aspects of pain perception, such as the sensory, emotional, and cognitive dimensions of pain (10–12). Moreover, pain perception is not solely determined by sensory input but also by psychological factors. The prolonged pain experiences tend to induce emotional and cognitive impairments, such as anxiety, depression, and memory loss (12–14). Therefore, it is important to investigate the mechanisms of pain modulation and to use effective strategies for early control of this irksome phenomenon.

A decrease in tissue pH is observed following inflammation, ischemia, as well as infections, while the physiological tissue pH range typically falls between 7.35 and 7.45 (15, 16). Extracellular acidification can sensitize widely distributed acid-sensitive sensory neurons, making them more responsive to pain signals (13). In one article, repeated intramuscular injection of acidic saline into unilateral hindlimb muscles triggered hyperalgesia of the paw in rodents. This kind of acid-induced inflammation in the hindlimb activates pain-sensing receptors located at primary afferent fibers, transmitting pain signals to the spinal cord and ultimately to higher brain centers, including the hindlimb somatosensory cortex (S1HL) (17, 18). S1HL is organized in a layer-specific manner and has a bidirectional role in modulating subjective sensory information. Layer 6 (L6) of S1HL activation increases somatosensory sensitivity and evokes spontaneous nocifensive behavior, whereas L5 activation exerts an antinociceptive effect in inflammatory pain models (6, 19, 20). Moreover, inhibition of glutamatergic neuronal circuits from the ventral posterolateral nucleus of the thalamus to the S1HL reversed allodynia in the mice model of chronic pain (21). These studies were usually restricted to neurons of S1HL, although astrocytes exerting local regulatory activity in response to neuronal signaling molecules should be also considered as modulators of the pain pathway.

Astrocytes, which are abundant in the central nervous system (CNS), have been found to play a role in regulating neurotransmitter release and thereby inflammation. It has been reported that astrocytes are not merely passive supporting elements of nociceptive neurons, but actively participate in pain processing (22). In one study, selective activation of astrocytes in S1 reversed the aberrant pain-like behavior induced by partial sciatic nerve ligation (23). The function of cortical astrocytes in pain modulation is important for finding feasible approaches for pain management.

Due to concerns about the addiction and overdose of classical opioid analgesics, there is a continued emphasis on finding non-opioid alternatives for pain management. Acupuncture has been used to relieve acute and chronic pain for thousands of years in China. In the last few decades, electro-acupuncture (EA), namely electrical stimulation via the acupuncture needles, has frequently been used in clinics and has been proven effective in pain disorders (24, 25). Recent studies have revealed that the analgesic effect of EA in neuropathic pain took place probably through inhibiting astrocytes and microglia in the spinal dorsal horn (26), and through activating inhibitory neural circuits in the S1 (27). Therefore, we sought to investigate the potential role of EA and cortical astrocytes in acid-induced acute pain.

In this study, we assumed that EA analgesia might act by modulating astrocytic activity in the S1 to alleviate the acid-induced pain in rodents. To test this hypothesis, we first confirmed the analgesic effects of EA in the pH 6.0 phosphate-buffered saline (PBS)-induced pain model in male rats. Then, we performed selective activation or inhibition of astrocytes in the S1HL by chemogenetics, proving the moderate analgesic effects of astrocytic activation. Furthermore, EA was used after activating or inhibiting astrocytic functions, suggesting the positive correlation between astrocytes and EA. In addition to reducing thermal hyperalgesia in rats, EA upregulated the expression of glial fibrillary acidic protein (GFAP; one reactive astrocyte marker) in the S1HL. These results provide a novel view on the involvement of EA and S1HL astrocytes in early pain control.

## 2 Materials and methods

### 2.1 Animals

All experimental procedures were conducted following the National Institutes of Health (NIH) Guidelines for the Care and Use of Laboratory Animals and approved by the Animal Ethics Committee of Chengdu University of Traditional Chinese Medicine (protocol code, DC1237, 01 January 2019). The experiments were performed on male Sprague–Dawley (SD) rats (weighing 220–250 g) aged 6–8 weeks that were purchased from Chengdu Dossy Experimental Animals Co., Ltd. Animals were housed at standard laboratory conditions (24 ± 2°C room temperature and 65 ± 5% relative humidity on 12/12 h conventional light–dark cycles) and fed with standard laboratory chow and tap water *ad libitum*. After adaptive domestication for 1 week, mice were divided into different groups based on random numbers generated by the IBM SPSS Statistic 25 software, and assigned to individual mice.

### 2.2 EA stimulation

**Figure 1:**
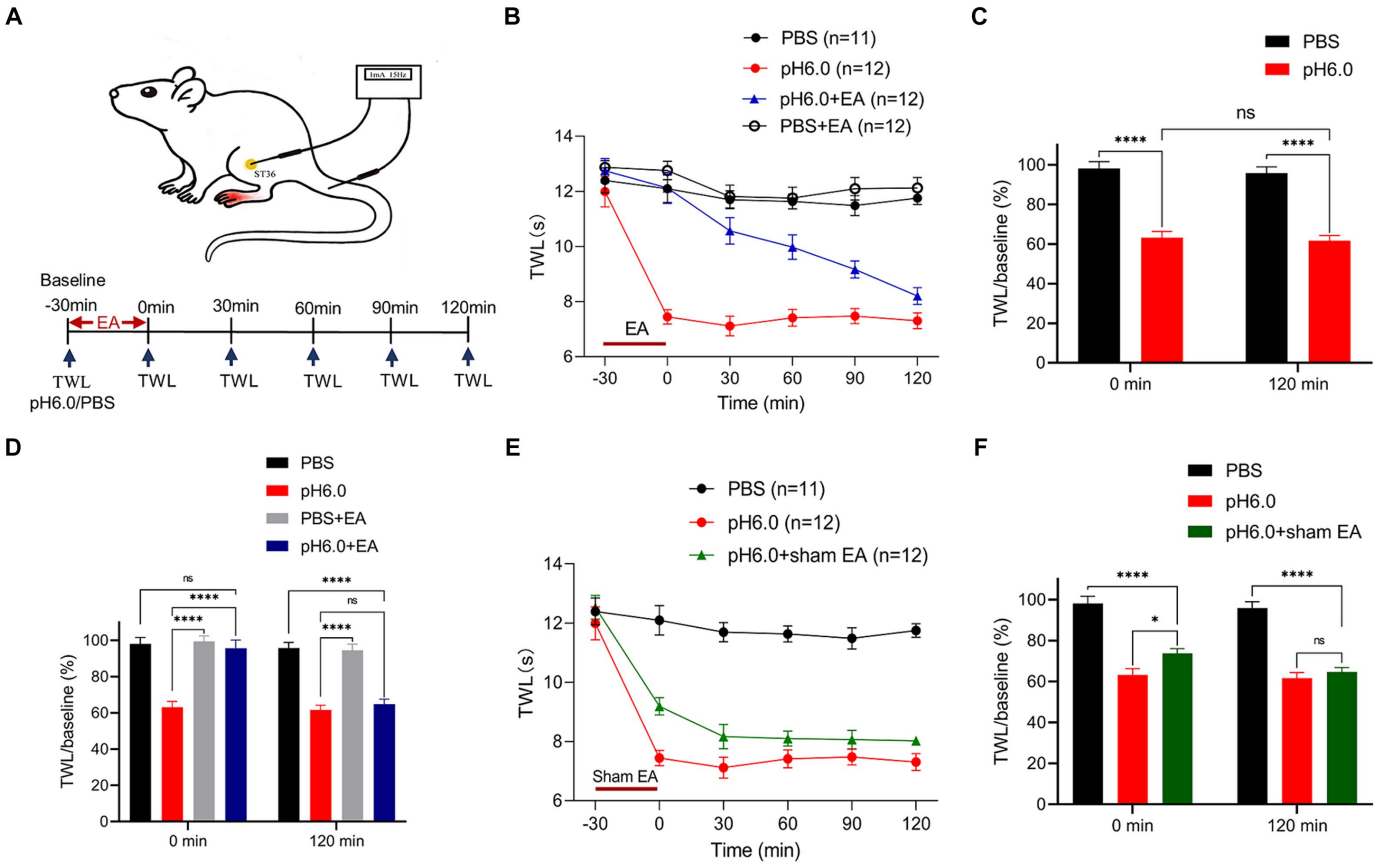
Electroacupuncture (EA) stimulation had an analgesic effect on pH 6.0-induced pain as measured in the left hind paw of rats. **(A)** Schematic diagrams showing the location of the “Zusanli” acupoint (ST36) and EA treatment in the rat, as well as the experimental timeline. **(B)** Time-dependent changes of thermal withdrawal latency (TWL) values after the application of normal or pH 6.0 phosphate-buffered saline (PBS) into the left hind paw and accompanying EA stimulation at ST36. Ratios of TWL values measured at the indicated times and at baseline (−30 min; TWL/baseline) at the 0- and 120-min time points without **(C)** and with EA **(D)**. **(E)** Time-dependent changes of the TWL values after sham EA stimulation. **(F)** TWL/baseline ratios at the 0-min and 120-min time points after sham EA stimulation. In this and in all further Figs, means ± S.E.M. values were calculated from measurements made in the indicated number of animals, as shown in brackets. *p ≤ 0.05; **p ≤ 0.01; ***p ≤ 0.001; ****p ≤ 0.0001.

EA stimulation was administered by using an electroacupuncture apparatus (HANS-200A Acupoint Nerve Stimulator, Nanjing Jisheng Medical Technology Co., Ltd., Jiangsu, China) at roughly the same time of the day (10:00 a.m. to 12:00 p.m.). One stainless-steel acupuncture needle (0.25 × 25 mm, Hwato-Med. Co., Jiangsu, China) was inserted into the left “Zusanli” acupoint (ST36), located about 6 mm down from the left fibular head, with a depth of 5–8 mm (Figure 1A). Another needle was applied to the region without acupoints, namely the stump of the tail. The position of ST36 in rats corresponds anatomically to its location in humans. For sham EA at ST36, the needle was inserted 2–3 mm deep into the skin dermal tissue and left there for 30 min, without any electrical stimulation. In the case of EA, the negative output of the stimulator was connected to the needle at ST36, and the auxiliary needle was connected to the positive output of the stimulator. The electrical current range was set at 1 mA, with a frequency of 15 Hz for 30 min (Figure 1A). The rats were immobilized by a self-made device during treatment (28).

### 2.3 Acid-induced pain models and behavioral testing

The pH of PBS (Sigma-Aldrich, Shanghai, China) was adjusted to 6.0 using acetic acid (Sigma-Aldrich) and sodium hydroxide (Sigma-Aldrich). 100 μL of PBS (pH 6.0) was injected into the left hind paw of SD rats to induce plantar hyperalgesia. Plantar pain threshold was determined as thermal withdrawal latency (TWL) by using a Thermal Stimuli Instrument (PL-200, Techman Software Co., Chengdu, China). The plantar surface of the left hind paw responds to thermal laser stimulation so that withdrawal, shaking, or licking of the left hind foot becomes apparent. Each rat was tested six times, with intervals of 5 min, every 30 min, including the following six points-in-time: −30 (baseline), as well as 0, 30, 60, 90, and 120 min. pH 6.0 PBS was injected at the point of 0 min (Figure 1A). All rats were placed separately into a transparent plastic enclosure (210 mm × 210 mm × 160 mm) on the surface of a vitreous platform (800 mm × 400 mm × 165 mm) for 30 min every day to get accustomed to the experimental conditions, 3 days before behavioral testing. All behavioral data were recorded by the same investigator who was blind to the experimental grouping. Rats with less than a 30% decrease in pain threshold at time-point 0-min were removed.

### 2.4 Stereotaxic surgery

**Figure 2:**
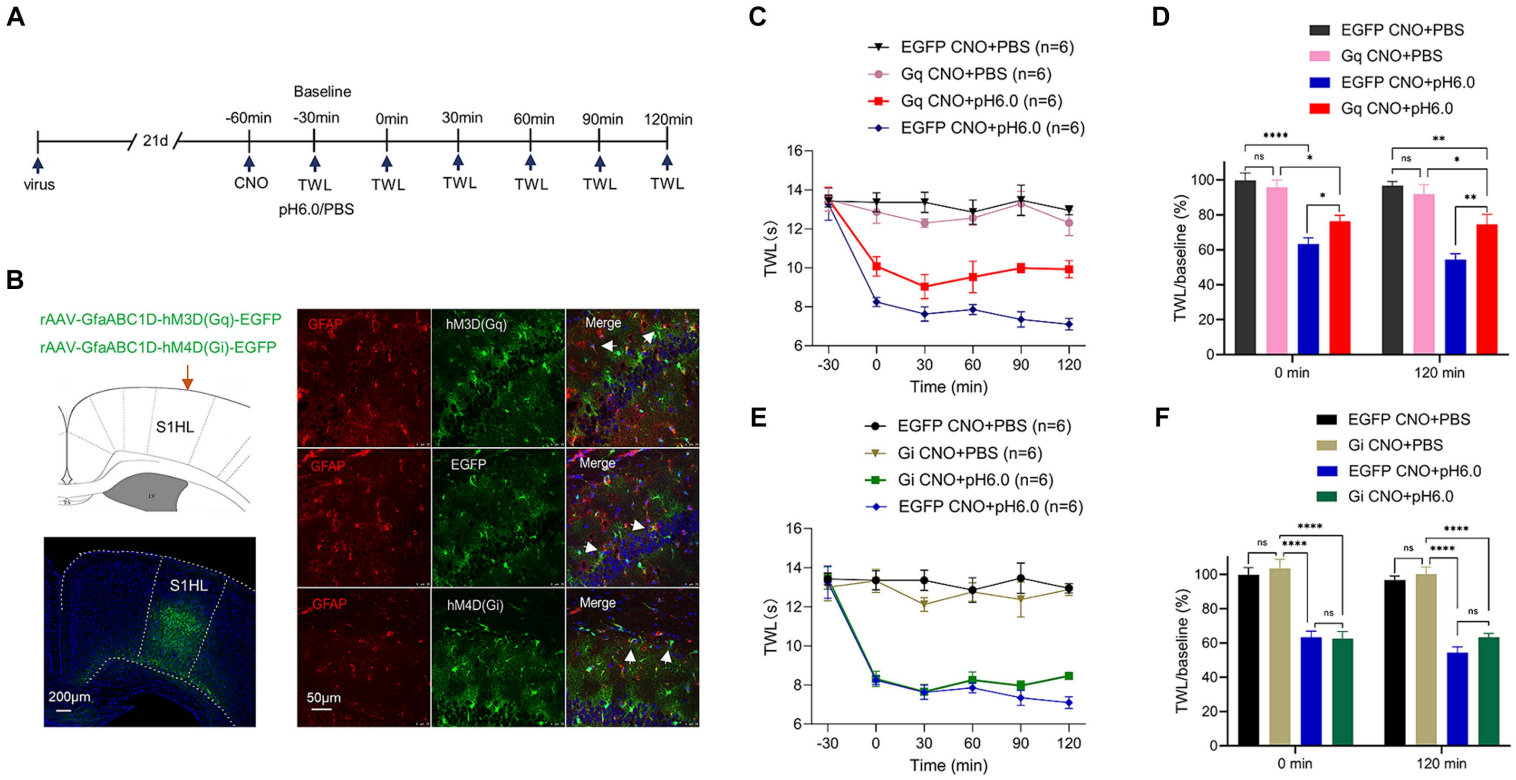
Chemogenetic activation of astrocytes in the hindlimb somatosensory cortex (S1HL) dampened pH 6.0-induced pain. **(A)** Diagram of the experimental time course. **(B)** Immunofluorescence results of viral expression in S1HL, and the co-location of the red GFAP biomarker of activated astrocytes, and the green chemogenetic protein, EGFP. Red fluorescence: GFAP; green fluorescence: hM3D (Gq), EGFP, or hM4D (Gi); blue fluorescence: DAPI. Arrowheads show co-staining of GFAP/hM3D (Gq), GFAP/EGFP, and GFAP/hM4D (Gi). **(C)** Change of TWL values after chemogenetic activation of astrocyte on pH 6.0-induced pain. **(D)** TWL/baseline ratios after chemogenetic astrocyte activation during normal or pH 6.0 PBS injection at the 0-min and 120-min time points. **(E)** Change of TWL values after chemogenetic inhibition of astrocytes on pH6.0-induced pain. **(F)** TWL/baseline values after chemogenetic astrocyte inhibition during normal or pH 6.0 PBS injection at the 0-min and 120-min time-points. *p ≤ 0.05; **p ≤ 0.01; ***p ≤ 0.001; ****p ≤ 0.0001.

The SD rats were anesthetized with isoflurane (5% for induction; 2% for maintenance; RWD Life Science, San Diego, CA, USA) and their head was fixed on a stereotaxic platform (RWD Life Science). 0.5 μL of adeno-associated virus (AAV) in a glass syringe was injected bilaterally into the S1HL (stereotaxic coordinates: AP -1.2 mm, ML ± 3.0 mm, DV -2.0 mm; see Figure 2B) at a rate of 0.05 μL/min with a microsyringe pump (RWD Life Science). An additional 10 min were allowed for diffusion and prevention of backflow. At the end of the surgery, 5 mg/kg enrofloxacin (RWD Life Science) was administered subcutaneously to the animals to prevent postoperative infection, and all animals were placed on heating pads (37°C) during surgery to keep their body temperature stable.

### 2.5 Chemogenetic manipulation

For chemogenetic manipulation, the following viruses were used in this study: rAAV-GfaABC1D-hM4D(Gi)-EGFP (titer: 2.02 × 10^12^ VG/mL, AAV2/5), rAAV-GfaABC1D-hM3D(Gq)-EGFP (titer: 2.10 × 10^12^ VG/mL, AAV2/5), rAAV-GfaABC1D-EGFP (titer: 2.10 × 10^12^ VG/mL, AAV2/5, all from Brain Case, China). The specific application mode of the virus is documented in Supplementary Table 1. The expression of the virus was checked by immunofluorescence staining after all tests were finished.

At least 3 weeks were allowed to pass for the complete expression of the virus. The activity of astrocytes was modulated by the chemogenetic receptors hM3Dq (Gq) and hM4Di (Gi) (see Supplementary Table 1 for specific manipulations). Next, rats were intraperitoneally injected with 1 mg/kg of clozapine N-oxide (CNO; Sigma-Aldrich, Saint Louis, MO, USA) to activate Gq and Gi receptors and the baseline of TWL was measured 30 min later. The pH 6.0 PBS was injected into the left paw of the hindlimb after obtaining the TWL (baseline), which was determined subsequently every 30 min until the time point of 120-min (Figure 2A).

### 2.6 Immunofluorescent analysis

SD rats were anesthetized with 2% pentobarbital sodium (40 mg/kg; Sigma-Aldrich) and transcardially perfused with 200 mL of 0.9% NaCl followed by 4% paraformaldehyde (PFA). Their brains were prepared and fixed in 4% PFA for 24 h, then they were stored at −80°C after gradient dehydration. The collected tissues were embedded in Tissue-Tek OCT compound (Sakura Finetek, Umkirch, Germany) and cut into 15-μm-thick sections with a freezing microtome (CM1806, Leica, Zurich, Switzerland); then they were incubated with the following antibodies: Mouse anti-GFAP antibody (1: 200, Proteintech Group, Chicago, USA), Goat Anti-Mouse IgG H&L (1: 400, Bioss, Beijing, China), Goat Anti-Mouse IgG H&L/Cy3 (1: 400, Bioss). Images were acquired using a confocal laser scanning microscope (Zeiss LSM700, Oberkochen, Germany) and quantified using Image J software. The quantity of GFAP immunoreactivity was expressed as GFAP-positive area in percentage of the total area in which GFAP-labelled immunoreactivity was determined.

### 2.7 Statistical analysis

All values were expressed as Mean ± S.E.M. (standard error of means). The data were analyzed and plotted using IBM SPSS Statistic 25 and GraphPad Prism 9. The normality test was conducted in IBM SPSS Statistic 25; all data were normally distributed. Multiple comparisons of data were performed with GraphPad Prism 9. Multiple groups were compared by one-way ANOVA followed by the Bonferroni *post-hoc* test. The statistical significance was defined as follows: *p ≤ 0.05; **p ≤ 0.01; ***p ≤ 0.001; ****p ≤ 0.0001; *p ≤ 0.05 was considered statistically significant.

## 3 Results

### 3.1 EA relieves pH 6.0-induced thermal hyperalgesia

To assess the analgesic effects of EA, we used the plantar injection of pH 6.0 PBS in the left hindlimb of SD rats to establish an acid-induced pain model and used the TWL test for 120 min to measure the change of pain threshold during this time (Figure 1A). We found that every group (normal PBS, pH 6.0 PBS, PBS + EA, pH 6.0 + EA) showed a similar baseline of the TWL at the −30 min time-point (Figures 1B,E). The acidic PBS caused a pronounced fall in the TWL value, 30 min after injection to the left paw that remained stable for 120 min, although the low pH PBS was expected to become diluted constantly in the tissue; by contrast, the injection of PBS at a normal pH of 7.4 had no impact on the TWL (Figures 1B,C). The results suggested that plantar injection of pH 6.0 PBS induced acute thermal hyperalgesia, and the acid-induced pain model was successfully established.

EA treatment was applied to the ipsilateral acupoint ST36 for 30 min after injecting normal or acidic PBS (Figures 1A–D). EA abolished the effect of pH 6.0 PBS at 0 min and this antagonism continuously vanished throughout the following 120-min, when it finally was no longer apparent (Figure 1B). By contrast, EA had no effect on the TWL measured after the injection of normal PBS. Figure 1C shows that the application of pH 6.0 PBS had the same effect at 0 and 120 min when expressed as the ratio of TWL and its baseline value at these two time points. In contrast, the TWL ratio measured at 0-min did not change when EA was applied in combination with acidic PBS. Nonetheless, this effect of EA was only temporary, and completely disappeared at the 120 min time point (Figure 1D). We also investigated the effect of sham EA, and found that this treatment failed to alter the effect of the acidic PBS on the TWL (Figures 1E,F).

### 3.2 EA may enhance the expression of GFAP in S1HL

**Figure 3:**
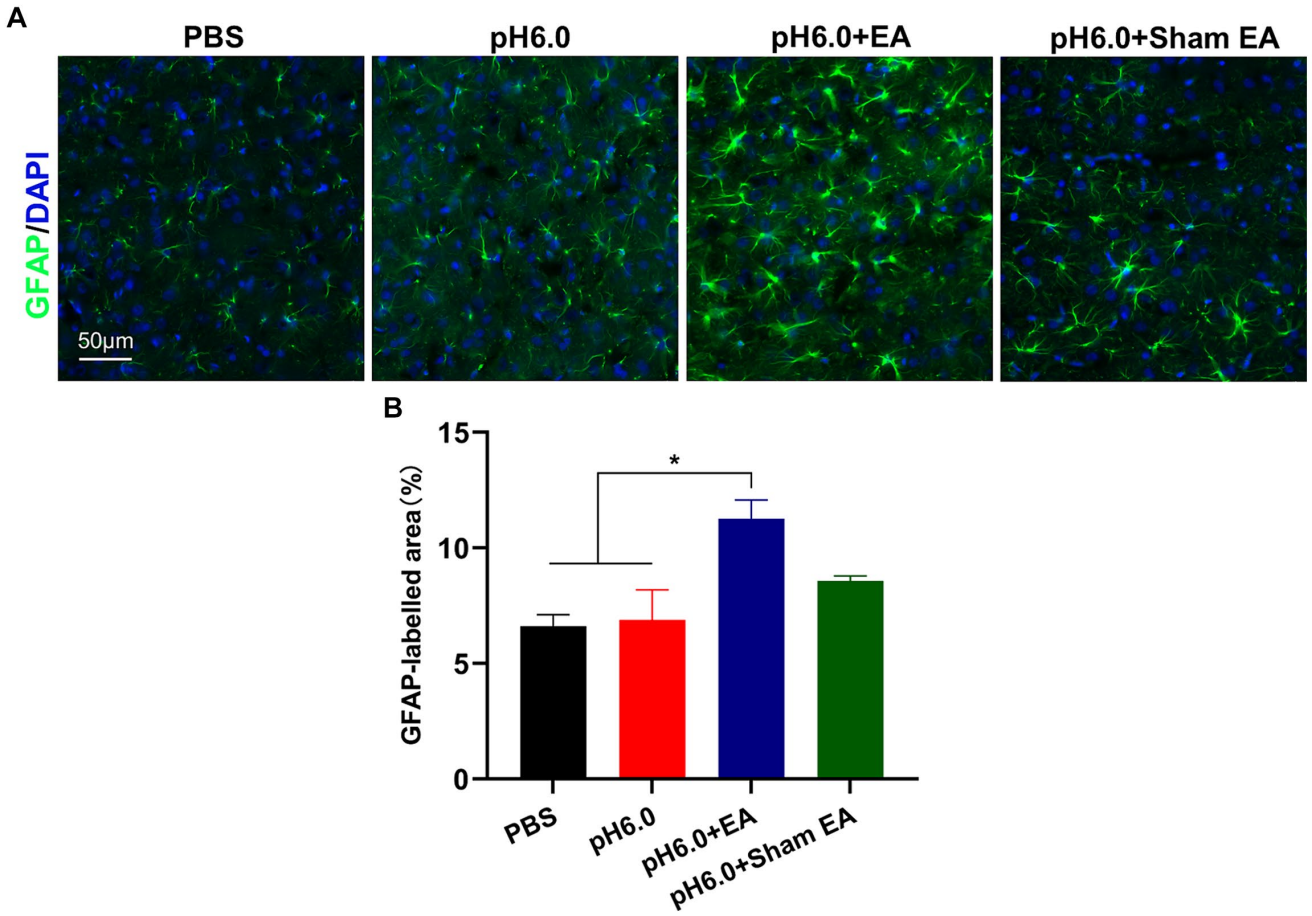
EA promoted astrocytes activation in the hindlimb somatosensory cortex (S1HL). **(A)** Immunofluorescence of GFAP expression of astrocytes after EA or sham EA in the contralateral (right) S1HL region. Green fluorescence: GFAP; blue fluorescence: DAPI. **(B)** The percentage of area with green fluorescence in relation to the total area in which GFAP immunoreatctivity was determined (*n* = 5).

GFAP is the most widely used biomarker of reactive astrocytes (29). To investigate the influence of EA treatment on astrocytic activation, we conducted immunofluorescence staining and quantified the expression of GFAP in the contralateral (right) S1HL region after injection of normal and acidic PBS, applied in combination of the latter with EA or sham EA (Figure 3A). The injection of pH 6.0 PBS into the left hind paw did not change the amount of GFAP (percentage of GFAP area; see Methods), when compared with that measured after the injection of normal pH PBS (Figures 3A,B). In contrast, the application of EA after low pH stimulation, markedly increased the amount of GFAP staining (Figure 3B), probably indicating the activation of contralateral astrocytes and the consequent development of astrogliosis. The combination of pH 6.0 PBS with sham EA had no comparable effect, although the tendency of the amount of GFAP staining to increase might be due to the mechanical stimulation of subcutaneous tissue in the acupoint by the needle, without delivering an accompanying electrical current.

### 3.3 Astrocytic activation of S1HL alleviates pH 6.0-induced plantar pain

We next examined whether astrocytes regulated the pH 6.0-induced pain. Immunmohistochemistry showed that the chemogenetic receptors were in fact expressed in GFAP-positive astrocytes in the S1HL region within the different experimental groups (Figure 2B).

The graphs illustrate that the TWL baselines were not modified when either Gq or Gi receptors were expressed in rat brains and were afterwards activated by intraperitoneal injection of CNO (1 mg/kg) (Figures 2C,E). The stimulation of S1HL astrocytes via Gq activation failed to restore the normal TWL but slightly prolonged the time until the onset of the pH 6.0-induced paw withdrawal from thermal stimulation (Figure 2C). In partial disagreement with these findings, abolishing the influence of S1HL astrocytes by Gi-mediated inhibition did not produce any change of TWL in rats caused by acidic PBS (Figure 2E). The respective control measurement in rats whose S1HL region was infected with the EGFP-carrying rAAV, which however lacked the Gq or Gi components, did not interfere with the pain-inducing effect of normal or acidic PBS (Figures 2C,E). This becomes still better visible, when the percentage TWL/baseline ratios are being considered, as documented in the Figures 2D,F.

### 3.4 Selective control of S1HL astrocyte regulates the analgesic effect of EA

We have proven that the pH 6.0-induced hyperalgesia could be strongly suppressed by EA stimulation and rather moderately by astrocytic activation via Gq receptors alone. Considering the positive link between EA and astrocytes that was found in previous work (26, 27) we directed our attention to the relationship between EA-induced analgesia and astrocytic functions. To illustrate the involvement of the S1HL astrocytes in the pain control of EA stimulation, EA was applied for 30 min after activating the chemogenetic receptors Gq or Gi by CNO in the pH 6.0-induced pain model of rats.

**Figure 4:**
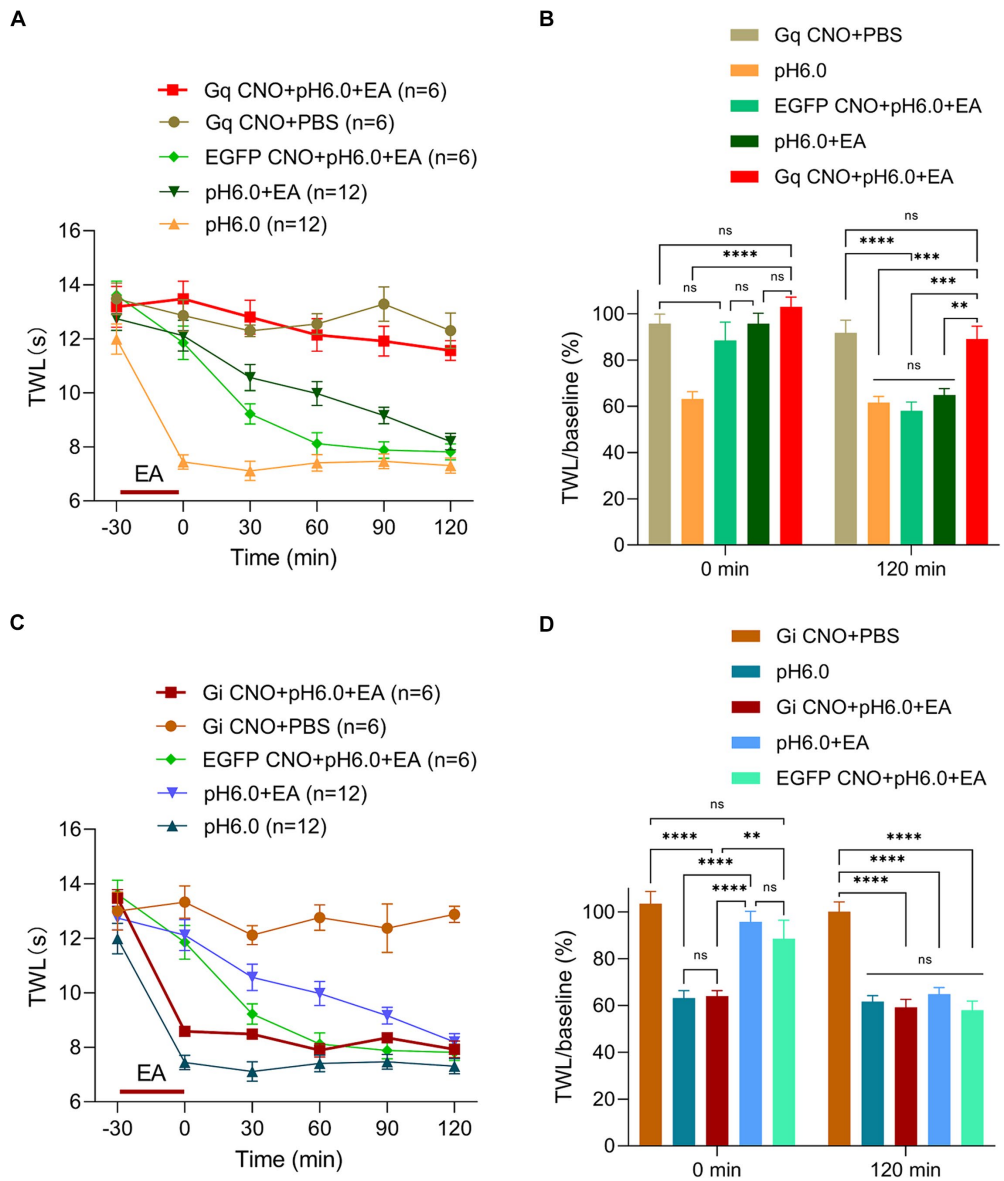
Astrocytes activation in S1HL plays a key role in EA analgesia. Experimental procedures were as described in Figure 2A and EA was applied from −30-min to 0-min, **(A)** Change of TWL values after chemogenetic astrocytes activation via hM3Dq and subsequent EA stimulation. **(B)** TWL/baseline ratios after chemogenetic astrocytes activation and EA stimulation during normal or pH 6.0 PBS injection at the 0-min and 120-min time points. **(C)** Change of TWL values after chemogenetic astrocytes inhibition via hM4Di and subsequent EA stimulation applied to the St36 acupoint on pH 6.0-induced pain. **(D)** TWL/baseline values after chemogenetic astrocytes inhibition and EA stimulation during normal or pH 6.0 PBS injection at the at 0-min and 120-min time-points.

The respective TWLs show that EA applied to rats with expressed Gq receptors in cortical astrocytes and added CNO, strongly potentiated the effect of EA (Figure 4A). This was evident also when the TWL/baseline ratios were taken into consideration (Figure 4B). To further elucidate the role of S1HL astrocytes, we repeated our experiments after inhibiting these astrocytes through the stimulation of their expressed Gi receptors by CNO. We found that inhibition of the activity of S1HL astrocytes conspicuously counteracted the analgesic effect of EA, whereas CNO administration to rats without available chemogenetic receptors (only EGFP present) was not able to remove this effect of EA (Figure 4C). As already pointed out, it is quite clear that the analgesic effect of EA was maximal at the time point of 0-min, and then gradually diminished until it disappeared at the time point of 120-min. Correspondingly, the antagonistic effect of astrocytic inhibition via Gi on EA effects was the largest at 0-min and totally vanished at 120-min (Figure 4D). This observation emphasized the involvement of astrocytes in EA-induced analgesia of acidic thermal hypersensitivity. Thus, the Gi-mediated inhibition of S1HL astrocytes suppressed, whereas the Gq- mediated activation potentiated the analgesic effect of EA against acid-induced pain.

## 4 Discussion

The main finding of our study is that EA applied to ST36 alleviated acid-induced muscular and cutaneous hyperalgesia through the modulation of S1HL astrocytes. Specifically, S1HL astrocytes were not, or only minimally involved in the sensation of peripheral acidic pain in rats, while these astrocytes unequivocally participated in EA-induced analgesia. Thus, astrocytes in the somatosensory cortex appear to be important stations in the pain pathway, that contribute via astrocyte-neuron interaction to EA-induced analgesia in acidic hyperalgesia.

It has been proven that tissue acidosis causes strong pain in humans and rodents (15, 16, 30). The present study utilizes a rat model in which acidic PBS was injected subcutaneously. This injection immediately decreased the TWL by approximately 40% below baseline; apparently a rapid drop of tissue pH led to localized hyperalgesia in rats. Previous investigations showed that plantar injection of pH 6.0 activated both acid-sensing ion channels 3 (ASIC3) and transient receptor potential vanilloid 1 (TRPV1) channel in rats, while mainly ASIC3 contributed to a lowering of the pain threshold (28, 31–33). ASIC3 is found to be expressed predominantly in peripheral sensory neurons and has been reported to be associated with acid-induced primary and secondary hyperalgesia (32, 34). Extracellular acidification would activate ASIC3 on primary sensory fibers to transmit the nociceptive signal through the spinal cord to S1 (26). Whether cortical astrocytes are involved in this pathway remains still unclear, especially because a few studies have reported, in contrast to our own findings, that reactive astrocytes facilitate pain transmission (22, 35). In our experiments, however, pH 6.0 injection did not upregulate the immunoreactivity of GFAP in S1HL, suggesting that acid-induced plantar pain cannot trigger the activation of cortical astrocytes. Moreover, chemogenetic inhibition of astrocytes failed to dampen plantar hyperalgesia either. Hence, our results provide evidence for the idea that cortical astrocytes participate probably only to a minor extent in the processing of peripheral ASIC3-mediated pain.

On the other hand, we found that Gi-mediated inhibition of S1HL astrocytes reversed the analgesic effect of EA stimulation, applied to ST36. By contrast, the Gq-mediated activation of cortical astrocytes had the opposite effect, and caused massive potentiation of the EA-induced analgesia. Gq-coupled receptors in astrocytes produce a sustained effect for more than 120 min, when activated by CNO (36); thus, stimulated S1HL astrocytes could produce a relatively long-lasting analgesic effect in combination with EA-induced analgesia. One astrocyte can contact thousands of synapses, which enables astrocytes to regulate local neurotransmission and extracellular microenvironment in the CNS (37, 38). For example, it has been reported that activation of S1 astrocytes by chemogenetics could reverse allodynia-like behavior previously established by partial sciatic nerve ligation. The underlying mechanism of this effect is that activation of S1 astrocytes results in synaptic plasticity of cortical circuits (23). Moreover, astrocyte activation has been found to block nociceptive transmission through the activation of endogenous adenosinergic mechanisms in the spinal cord (39). Adenosine, activating the adenosine A1 receptors (A1Rs) produces suppression of neuronal responses (40), resulting in inhibition of inflammation and pain (15, 41). Meanwhile, it is well established that astrocytes are the key regulators of extracellular levels of adenosine in the CNS (42, 43). Hence, it is likely that activated cortical astrocytes produce analgesia via adenosinergic modulation of cortical circuits. Future work should use astrocyte-specific conditional KO model mice to allow a better understanding of the biological role of adenosine in inflammation and pain.

The interaction between astrocytes and acupuncture is a frequented area of research. EA is commonly recognized to inhibit astrocyte activation and thereby to cause analgesia (44, 45). Here, we observed that EA could indeed relieve acid-induced pain, but this effect was reversed by the chemogenetic inhibition of astrocytic activity in S1HL. Thus, the astrocytic activation is essential for EA therapy of acid-induced hyperalgesia. The activation of S1 astrocytes by EA stimuli delivered to ST36 have been confirmed by the measurement of calcium transients in this area of the brain (46). In the brain cortex, activated astrocytes release ATP which is rapidly hydrolyzed to adenosine and thereby regulates synaptic transmission (47). Moreover, EA is reported to trigger the release of endogenous adenosine and to activate adenosine A1Rs at sensory nerve terminals to relieve inflammatory pain (42, 48, 49). We thus speculate that endogenous adenosine of cortical astrocytes may mediate EA-induced analgesia in acid-induced pain. The present study also showed that the combined use of EA and chemogenetic astrocyte activation potentiated the suppression of acid-induced acute hyperalgesia by EA. This brings us to the layer specificity of the cortex, as different layers may have opposite effects on pain control (6). Hence, ST36-mediated EA analgesia may rely on the astrocytic activation of one of the layers in S1HL, with some likelihood that of L5. However, in our experiments, the expression of Gq protein was observed in multiple layers of S1HL. Presently, it is not possible to decide which layer plays a decisive role in EA-mediated stimulation of S1HL astrocytes. It would be necessary to confine the expression of the Gq-carrying virus to individual layers of the S1HL to find out which cortical layer has the highest significance for EA analgesia. These finding may explain the discrepancy between our results and those of other groups of researchers (26, 27).

The present study further supports the involvement of astrocytic functions in acupuncture analgesia. It is important to note that the exact mechanisms through which EA influences astrocytic activation and acid-induced pain are not fully understood, and results may vary depending on the specific context and methodology of the studies. A limitation of our experiments is that we used only male rats, yet female rodents and humans are known to exhibit some differences in pain biology (50).

In conclusion, our data provide evidence for the assumption that cortical astrocytes exert an essential function in EA-induced analgesia of acid-induced pain. Combining EA stimulation with astrocytic activation by pharmacological means could be a viable approach for the early management of acute pain.

## Data availability statement

The raw data supporting the conclusions of this article will be made available by the authors, without undue reservation.

## Ethics statement

The animal studies were approved by the Animal Ethics Committee of Chengdu University of Traditional Chinese Medicine. The studies were conducted in accordance with the local legislation and institutional requirements. Written informed consent was obtained from the owners for the participation of their animals in this study.

## Author contributions

QY: Data curation, Formal analysis, Investigation, Methodology, Writing – original draft. JL: Data curation, Formal analysis, Investigation, Methodology, Writing – original draft. W-JR: Investigation, Methodology, Writing – review & editing. YZ: Investigation, Methodology, Writing – review & editing. TW: Investigation, Methodology, Writing – review & editing. PR: Resources, Writing – review & editing. H-YY: Resources, Writing – review & editing. PI: Conceptualization, Funding acquisition, Supervision, Writing – original draft, Writing – review & editing. YT: Conceptualization, Funding acquisition, Supervision, Writing – original draft.
