# Supplementary material for: Astrocyte activation in hindlimb somatosensory cortex contributes to electroacupuncture analgesia in acid-induced pain
Source: Front Neurol. 2024 Apr 3;15:1348038. doi: 10.3389/fneur.2024.1348038 (PMC11021577; doi:10.3389/fneur.2024.1348038)
Supplement: Supplementary file 1 [file Table_1.pdf]

Supplementary table

Tab.1. Treatments to different groups of rats in this study.

Group name	Virus injection	Virus function	Treatment	Number of rats
PBS	/	/	plantar injection of normal PBS	11
pH 6.0	/	/	plantar injection of normal acidic PBS	12
PBS+EA	/	/	EA stimulation after plantar injection of normal PBS	12
pH 6.0+EA	/	/	EA stimulation after plantar injection of acidic PBS	12
pH 6.0+sham EA	/	/	sham EA stimulation after plantar injection of acidic PBS	12
EGFP CNO+PBS	rAAV-GfaABC1D-EGFP	control	plantar injection of normal PBS after CNO administration for 30 min	6
EGFP CNO+ pH6.0	rAAV-GfaABC1D-EGFP	control	plantar injection of acidic PBS after CNO administration for 30 min	6
Gq CNO+PBS	rAAV-GfaABC1D-hM3D(Gq)-EGFP	astrocyte activation	plantar injection of normal PBS after CNO administration for 30 min	6
Gq CNO+ pH6.0	rAAV-GfaABC1D-hM3D(Gq)-EGFP	astrocyte activation	plantar injection of acidic PBS after CNO administration for 30 min	6
Gi CNO+PBS	rAAV-GfaABC1D-hM4D(Gi)-EGFP	astrocyte inhibition	plantar injection of normal PBS after CNO administration for 30 min	6
Gi CNO+ pH6.0	rAAV-GfaABC1D-hM4D(Gi)-EGFP	astrocyte inhibition	plantar injection of acidic PBS after CNO administration for 30 min	6
EGFP CNO+pH6.0+EA	rAAV-GfaABC1D-EGFP	control	plantar injection of acidic PBS after CNO administration for 30 min, then apply EA	6
Gq CNO+pH6.0+EA	rAAV-GfaABC1D-hM3D(Gq)-EGFP	astrocyte activation	plantar injection of acidic PBS after CNO administration for 30 min, then apply EA	6
Gi CNO+pH6.0+EA	rAAV-GfaABC1D-hM4D(Gi)-EGFP	astrocyte inhibition	plantar injection of acidic PBS after CNO administration for 30 min, then apply EA	6
